# Maternal obesity, environmental factors, cesarean delivery and breastfeeding as determinants of overweight and obesity in children: results from a cohort

**DOI:** 10.1186/s12884-015-0518-z

**Published:** 2015-04-15

**Authors:** Daniel S Portela, Tatiana O Vieira, Sheila MA Matos, Nelson F de Oliveira, Graciete O Vieira

**Affiliations:** Federal University of Recôncavo da Bahia, CEP 44.570-000 Santo Antônio de Jesus, Bahia Brazil; State University of Feira de Santana, Feira de Santana, Bahia Brazil; Federal University of Bahia, Salvador, Bahia Brazil

**Keywords:** Breastfeeding, Overweight, Obesity, Child, Caesarean delivery

## Abstract

**Background:**

Overweight and obesity are a public health problem with a multifactorial aetiology. The objective of this study was to evaluate risk factors for overweight and obesity in children at 6 years of age, including type of delivery and breastfeeding.

**Methods:**

This study relates to a cohort of 672 mother-baby pairs who have been followed from birth up to 6 years of age. The sample included mothers and infants seen at all ten maternity units in a large Brazilian city. Genetic, socioeconomic, demographic variables and postnatal characteristics were analyzed. The outcome analyzed was overweight and/or obesity defined as a body mass index greater than or equal to +1 z-score. The sample was stratified by breastfeeding duration, and a descriptive analysis was performed using a hierarchical logistic regression. *P-values* of <0.05 were considered significant.

**Results:**

Prevalence rates (PR) of overweight and obesity among the children were 15.6% and 12.9%, respectively. Among the subset of breastfed children, factors associated with the outcome were maternal overweight and/or obesity (PR 1.92; 95% confidence interval “95% CI” 1.15–3.24) and lower income (PR 0.50; 95% CI 0.29–0.85). Among children who had not been breastfed or had been breastfed for shorter periods (less than 12 months), predictors were mothers with lower levels of education (PR 0.39; 95% CI 0.19–0.78), working mothers (PR 1.83; 95% CI 1.05–3.21), caesarean delivery (PR 1.98; 95% CI 1.14 – 3.50) and maternal obesity (PR 3.05; 95% CI 1.81 – 5.25).

**Conclusions:**

Maternal obesity and caesarean delivery were strongly associated with childhood overweight and/or obesity. Lower family income and lower levels of education were identified as protective factors. Breastfeeding duration appeared to modify the association between overweight/obesity and the other predictors studied.

## Background

Overweight and obesity are a public health problem in both developed countries and those in development [1]. Prevalence rates of excess weight in Brazil have been increasing over recent decades [2].

Studies performed in Brazil at the end of the last century have shown a prevalence varying from 10.8% to 26.2% for childhood overweight, and 7.3% to 8.5% for childhood obesity [3,4]. A decade later, research has shown that the prevalence of childhood overweight varies from 15.3% to 34% [5,6].

The multifactorial aetiology of obesity is consensus in the literature, involving genetic, environmental, socioeconomic and behavioral factors [4,7]. In view of the difficulty of treating the condition, identification of risk factors, and most importantly those that can be modified, provides epidemiological tools of fundamental importance for planning intervention measures with a positive impact on public health.

The literature discusses the association between factors present in the fetal period, neonatal period, and first months of life with childhood and adult diseases [8-12]. Many studies suggest that breastfeeding may be a protective factor against childhood obesity [6,11,13-16]. The lower protein content of breast milk is related to a lower risk of obesity in childhood. Furthermore, the practice of breastfeeding has a protective effect through the protein quality, for example the alpha-lactoalbumin [17], the auto regulation of volume ingested, and the improved acceptance of healthier foods [13,18]. However, some researchers question the protection of breast milk against childhood obesity [19].

Similarly, other determinants of risk in earlier phases of life are currently being studied, for example, caesarean deliveries. However, studies investigating this association are scarce [8,20].

The objective of this study was to evaluate the association between environmental and genetic risk factors, type of birth, and overweight and/or obesity at 6 years of age.

## Methods

### Study design

This was a cohort study conducted in the city of Feira de Santana, which is 108 km from the state capital of Bahia, in Northeast Brazil.

### Population and study sample

The children enrolled on this study are part of a cohort that began in April 2004, comprising of all live births at all ten maternity units in the municipal district.The following exclusion criteria were used in this study: mothers who were separated from their children by the justice system (for example, imprisoned); children with diseases that contraindicate breastfeeding; those who live in locations that present a risk to the safety of the interviewer (for example, due to drug trade, prostitution).The sample size calculation for this study was based on a 6.0% estimated prevalence of overweight among children under 5 years old in the Northeast of Brazil [21], a level of precision of around 1.25% and 95% confidence, which resulted in an estimated sample of 526 children. This figure was increased by 30% to cover predicted losses, resulting in a final sample size calculation of 684 children. However, data from 672 children followed in the cohort from birth to 72 months were actually analyzed.

### Data collection

Questionnaires were administered by trained health professionals. The data analyzed were collected during the first 72 hours after delivery, during monthly home visits for the first six months of the children’s lives and during interviews conducted 9, 12, 36 and 72 months after the birth of each child.

### Variables

Body weights of mothers and their children were measured in triplicate at the 72-month home visit, using a digital balance with precision of 100 gram and a maximum capacity of 150 kg. Height was measured using a portable and dismountable stadiometer with a built-in platform and a maximum height of 216 cm.

Anthropometric status was assessed using body mass index (BMI) at six years of age, calculated using World Health Organization’s (WHO) Anthro Plus software, which uses the WHO growth curves as a reference [22,23]. Children were classified by nutritional status as underweight or normal weight if BMI was below a z-score of +1 or as overweight or obese if BMI was greater than or equal to a z-score of +1.

The variable of mother’s educational level was dichotomized with a cut off of 8 years, equivalent to middle school in Brazil, as this is the cut off commonly used by other Brazilian studies. The variable of mother’s age was dichotomized between adolescence (<20 years) and adult. Family income and sedentary activities were dichotomized according to criteria of convenience, based on measures of central tendency and dispersion.

The remaining variables analyzed were subdivided into three different groups, as follows, 1. socioeconomic and demographic characteristics: mother’s educational level, mother’s age, family income, mother was going out work at time of birth; 2. genetic characteristics: maternal BMI; 3. children’s neonatal characteristics: type of delivery, sex, birth weight, gestational age, and postnatal factors, as their lifestyle habits at six years of age, such as physical activity at school; total time spent daily in sedentary activities such as watching television, playing videogames or using a computer; sports outside of school; playing ball games, riding a bicycle and buying school meals.

### Statistical analysis

Initially, sample characteristics were compared by breastfeeding in its original continuous scale using Student’s *t* test. Effect modifying factors were then evaluated and stratified by breastfeeding, and the Pearson’s chi-square test (x^2^) was used to analyze factors associated with overweight/obesity. For all tests, a *p-value* of <0.05 was considered significant.

**Figure 1:**
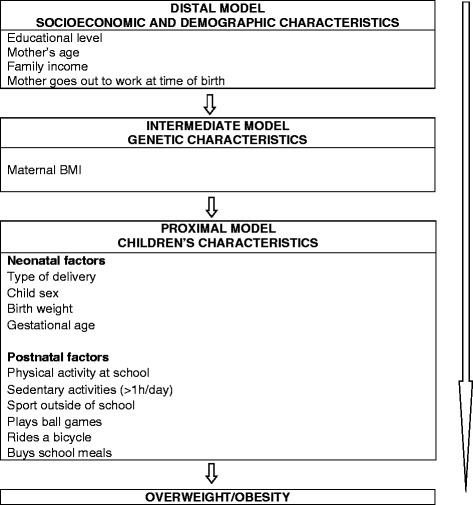
Diagram of hierarchical theoretical model of the determinants of overweight/obesity.

Logistic regression analysis was conducted on the two strata of children classified by breastfeeding duration, with insertion of variables according to a three-level hierarchical theoretical model (Figure 1), and variables distributed according to their proximity to the outcome: socioeconomic and demographic characteristics (model 1, distal level), genetic characteristics (model 2, intermediate level) and characteristics of the neonatal and postnatal period (model 3, proximal level).

All variables that attained a *p-value* of ≤ 0.10 in bivariate analysis were included in the hierarchical analysis. Variables that attained an intralevel statistical significance of 5% were then used to adjust the following level, and this procedure was repeated successively until inclusion of the last eligible variable in level 3, with definition of factors associated with overweight/obesity among children under six years to a significance level of 5%. The model’s fit was then tested. Analyses were conducted using SPSS 10.0 and R statistical software packages [24]. The same procedure was then conducted for two strata of children separated by breastfeeding duration.

### Ethical aspects

The study has been submitted and approved by the Ethics and Research Committee of the State University of Feira de Santana, Bahia, Brazil under Protocols 12/2003 and 077/2006. All women included in the study provided informed written consent.

## Results

### General characteristics of the sample

A total of 672 mother-child pairs were included in the analysis. Around half of the infants had already been weaned before the end of their 12 months (51.9%). At 72 months of age, 15.6% were overweight and 12.9% were obese. A high prevalence of maternal overweight and/or obesity (52.4%) was also observed.

Table 1 lists the characteristics of the sample by breastfeeding in its original continuous scale. Certain statistically significant differences between the two groups were detected: children who had been weaned before completing one year of life had mothers with lower educational levels, had higher family incomes, were more likely to have mothers who went out to work and engaged in more physical activity (Table 1).

**Table 1:** **Description of the sample and comparison of characteristics, by breastfeeding using Student’s** ***t*** **test**

**Variable analyzed**	**N (%)**	***p-value***
**Mother’s educational level**		
≤8 years of schooling	202 (30.1)	0.015
>8 years of schooling	470 (69.9)	
**Mother’s age (years)**		
<20	104 (15.5)	0.692
≥20	568 (84.5)	
**Family income**		
<2 times minimum wage	321 (47.8)	<0.000
≥2 times minimum wage	351 (52.2)	
**Mother goes out to work at time of birth**		
Yes	241 (35.9)	0.021
No	431 (64.1)	
**Mother’s BMI**		
Overweight/obesity	352 (52.4)	0.764
Underweight/normal weight	320 (47.6)	
**Type of delivery**		
Caesarean	327 (48.7)	0.123
Vaginal	345 (51.3)	
**Sex of child**		
Male	336 (50.0)	0.765
Female	336 (50.0)	
**Birth weight (grams)**		
<2,500	30 (4.5)	0.873
≥2,500	642 (95.5)	
**Gestational age**		
Preterm	26 (3.9)	0.620
Full term	646 (96.1)	
**Physical activity at school**		
Yes	179 (26.6)	0.035
No	486 (72.3)	
**Sedentary activities (>1 h/day)**		
Yes	621 (92.4)	0.369
No	51 (7.6)	
**Sport outside of school**		
Yes	79 (11.8)	0.091
No	593 (88.2)	
**Plays ball games**		
Yes	365 (54.3)	0.737
No	307 (45.7)	
**Rides a bicycle**		
Yes	468 (69.6)	0.939
No	204 (30.4)	
**Buys school meals**		
Yes	250 (37.2)	0.113
No	415 (61.8)	

N = number of children.

BMI = body mass index.

h = hour.

### Stratification of children by breastfeeding or weaned at 12 months

As shown in Table 2, we observed that weaning at 12 months is an interaction factor that alters the association between mother going out to work and overweight/obesity.

**Table 2:** **Bivariate analysis of factors associated with overweight/obesity stratified by breastfeeding at 12 months of age**

**Variables**	**Breastfeeding**			**Weaning**			**Interaction** ***p-value***
	**N**	**Overweight/obesity**		**N**	**Overweight/obesity**		
		**n (%)**	**PR (95% CI)**		**n (%)**	**PR (95% CI)**	
**Sociodemographic characteristics**							
**Mother’s educational level**							
≤8 years of schooling	120	20 (16.7)	0.57 (0.36 – 0.89)	82	13 (15.9)	0.42 (0.25 – 0.70)	0.291
>8 years of schooling	229	67 (29.3)		241	92 (38.2)		
**Mother’s age (years)**							
<20	56	8 (14.3)	0.53 (0.27 – 1.03)	48	11 (22.9)	0.67 (0.39 – 1.16)	0.663
≥20	293	79 (27.0)		275	94 (34.2)		
**Family income (multiples of minimum wage)**							
<2	194	34 (17.5)	0.51 (0.35 – 0.75)	127	31 (24.4)	0.65 (0.45 – 0.92)	0.462
≥2	155	53 (34.2)		196	74 (37.8)		
**Mother working outside the home at time of birth**							
Yes	108	30 (27.8)	1.17 (0.80 – 1.72)	133	59 (44.4)	1.83 (1.34 – 2.51)	0.050
No	141	57 (23.7)		190	46 (24.2)		
**Genetic characteristics**							
**Mother’s BMI**							
Overweight/Obesity	167	56 (30.8)	1.66 (1.13 – 2.45)	153	70 (41.2)	1.80 (1.28 – 2.53)	0.591
Underweight/Normal weight	176	31 (18.6)		170	35 (22.9)		
**Characteristics of the children**							
**Type of delivery**							
Caesarean	151	48 (31.8)	1.61 (1.12 – 2.33)	176	75 (42.6)	2.09 (1.45 – 3.00)	0.238
Vaginal	198	39 (19.7)		147	30 (20.4)		
**Sex of child**							
Male	165	37 (22.4)	0.83 (0.57 – 1.19)	171	55 (32.2)	0.98 (0.71 – 1.34)	0.520
Female	185	50 (27.2)		152	50 (32.9)		
**Birth weight (grams)**							
<2,500	17	3 (17.6)	0.70 (0.25 – 1.98)	13	2 (15.4)	0.46 (0.13 – 1.67)	0.588
≥2,500	332	84 (25.3)		310	103 (33.2)		
**Gestational age**							
Preterm	13	2 (15.4)	0.61 (0.17 – 2.21)	13	1 (7.7)	0.23 (0.04 – 1.52)	0.366
Full term	336	85 (25.3)		310	104 (33.5)		
**Physical activity at school**							
Yes	78	26 (33.3)	1.46 (0.99 – 2.15)	101	40 (39.6)	1.35 (0.98 – 1.85)	0.825
No	268	61 (22.8)		218	64 (29.4)		
**Sedentary activities (>1 h/day)**							
Yes	320	80 (25.0)	1.04 (0.53 – 2.03)	301	100 (33.2)	1.46 (0.67 – 3.21)	0.488
No	29	7 (24.1)		23	5 (22.7)		
**Sport outside of school**							
Yes	31	11 (35.5)	1.49 (0.89 – 2.48)	48	24 (50.0)	1.70 (1.21 – 2.38)	0.539
No	318	76 (23.9)		175	81 (29.5)		
**Plays ball games**							
Yes	178	41 (23.0)	0.86 (0.59 – 1.23)	187	57 (30.5)	0.86 (0.63 – 1.18)	0.973
No	171	46 (26.9)		136	48 (35.3)		
**Rides a bicycle**							
Yes	242	59 (24.4)	0.93 (0.63 – 1.37)	226	70 (31.0)	0.86 (0.62 – 1.19)	0.714
No	107	28 (26.2)		97	35 (36.1)		
**Buys school meals**							
Yes	109	36 (33.0)	1.54 (1.07 – 2.20)	141	55 (39.0)	1.42 (1.03 – 1.94)	0.821
No	137	51 (21.5)		178	49 (27.5)		

N = total number of children in “breastfeeding” or “weaning” group.

n = number of overweight and/or obesity children.

BMI = body mass index.

h = hour.

This explains the considerable difference in the prevalence ratios (PR) of overweight and/or obesity for the variable working mothers in the two strata (breastfed group PR 1.17; 95% CI 0.80-1.72; weaned group PR 1.83; 95% CI 1.28-2.50).

When associations between the three groups of characteristics (sociodemographic, genetic and children’s characteristics) and the outcome were analyzed for the two strata, certain factors were common to both groups (higher maternal educational level, higher family income, mothers with excess body weight, caesarean delivery and buying school meals), while working mothers and practicing sports outside of school were only associated with obesity among children who were weaned before 12 months (Table 2).

When the factors analyzed were adjusted using multivariate logistic regression analysis, it was found that having mothers with excess body weight was a predictor of risk of overweight/obesity at 6 years of age among children in both the strata. However, it is important to stress the fact that children breastfed for at least 12 months had a much lower risk (PR 1.92; 95% CI 1.15-3.24, Table 3) than those breastfed for shorter periods (less than 12 months) (PR 3.05; 95% CI 1.81-5.25, Table 4). In addition to maternal obesity, higher family income was a risk factor for overweight/obesity among breastfed children (Table 3). In contrast, for children who were weaned before 12 months, the predictors of overweight and/or obesity were as follows: higher maternal educational level, mother working outside the home at time of birth, and caesarean delivery (Table 4).

**Table 3:** **Logistic regression of characteristics associated with overweight/obesity among children breastfed for 12 months**

**Variables**	**Model 1-distal**	**p**	**Model 2-intermediate**	**p**	**Model 3-proximal**	**p**
	**PR (95% CI)**		**PR (95% CI)**		**PR (95% CI)**	
Income < 2 times minimum wage	0.41 (0.25 – 0.67)	<0.000	0.41 (0.25 – 0.68)	<0.000	0.50 (0.29 – 0.85)	0.011
Mother overweight/obese	----------------------	---	1.93 (1.17 – 3.24)	0.011	1.92 (1.15 – 3.24)	0.013

**Table 4:** **Logistic regression of characteristics associated with overweight/obesity among weaned children**

**Variables**	**Model 1-distal**	**p**	**Model 2-intermediate**	**p**	**Model 3-proximal**	**p**
	**PR (95%CI)**		**PR (95% CI)**		**PR (95% CI)**	
Mother’s educational level ≤8 years of schooling	0.39 (0.19 – 0.74)	0.006	0.33 (0.16 – 0.65)	0.002	0.39 (0.19 – 0.78)	0.009
Mother working outside the home at time of birth	2.01 (1.22 – 3.31)	0.006	2.34 (1.39 – 3.98)	0.001	1.83 (1.05 – 3.21)	0.032
Mother overweight/obese	----------------------	---	3.12 (1.86 – 5.32)	<0.000	3.05 (1.81 – 5.25)	<0.000
Caesarean delivery	----------------------	---	----------------------	---	1.98 (1.14 – 3.50)	0.016

## Discussion

### Childhood obesity: a public health problem

The results of this study reveal a prevalence of overweight and obesity of 28.5% (15.6% overweight and 12.9% obesity), similar to figures reported in other studies [2,25,26]. These results show the growth of the problem as the children age and invite discussion on the need for monitoring speed of weight gain from the start of life. Controlling childhood obesity is a current challenge for public health worldwide.

### Genetic and environmental aspects

The presence of obesity in adults is known to raise the risk of those adults having children who are also obese [2,10,27]. In this study, maternal obesity was associated with an increased risk of overweight/obesity, not only in breastfed children, but also in those who were weaned before 12 months.

Orera [28] found the risk children becoming obese increased 50% to 80% when the parents were obese. Marques-Lopes et al. [29] showed that the energy balance seemed to depend about 40% on genetic inheritance. It is worth pointing out that there is an overlap between heredity and dietary habits and behaviors shared by parents and children in the family environment.

Socioeconomic and demographic factors can act in parallel to the genesis of overweight and obesity in children and adults. In this study, some of these factors were associated with the outcome in both stratum of children studied. In those children breastfed for a longer period of time, lower family income was a protective factor. For those children who were weaned before 12 months, a lower level of mother’s schooling was a protective factor and mother going out to work was a risk factor.

Similarly, Oliveira et al. [30], researching school-age children in the same city as our study, found the same association between overweight and/or obesity with parental income and educational level. Costa et al. [31] evaluated private and public schools and in a city in Southeast Brazil and concluded that the risk of obesity was greater in those with a more favorable socioeconomic status. Other authors found a high prevalence of overweight and obesity in middle-class children in Northeast Brazil [4].

The results of this study contradict existing evidence in the literature on developed countries [32], where a greater family income and higher educational level were protective against overweight/obesity. However, no clear relationship is reported between maternal education and socio-economic status and childhood obesity [33-36]. Further research is necessary to better understand and confirm this association, which may vary depending on social context.

There is a tendency for developing countries to import the lifestyle of more socioeconomically developed countries. Greater household availability of processed food products in Brazil is positively and independently associated with higher prevalence of excess weight and obesity in all age groups in a cross-sectional study [37]. It is also relevant to mention studies that have called attention to differences between the dietary habits of breastfed children and those who were not breastfed, showing that prematurely weaned children have less healthy diets [18], which can contribute to increasing the risk of obesity at later ages [13,27].

Female participation in the employment market is a result of the new domestic economics of modern society. The absence of mothers from the home environment may have an impact on children’s nutritional status [38]. Some studies that have analyzed the associations between breastfeeding and overweight/obesity in children have discussed the increased risk of this outcome conferred on the children of mothers whose domestic roles have to compete with employment outside of the home [6,39]. Certain hypotheses can explain these results and are compatible with the results found in our study. Absence of mothers can alter children’s dietary habits, reducing breastfeeding duration, encouraging premature introduction of complementary foods and making it more likely that sweets and treats will be given to children to compensate for spending the day without their mothers [6].

### Caesarean delivery and childhood obesity

An interesting finding observed in this study was the association between type of delivery and overweight and/or obesity among children weaned before 12 months. The sample studied exhibited a high prevalence of caesarean delivery (47.6%), around three times higher than the rates recommended by the WHO [40]. The risk of overweight and/or obesity was two times higher in the group of children born from a caesarean delivery (PR 1.98; 95% CI 1.14-3.50) than among those born by vaginal delivery. This association has so far been the subject of little discussion in the literature, but similar data have recently been reported in other studies. Goldani et al. [20] describe PR of obesity of 15.2% in adults aged 23 to 25 who were born by caesarean delivery (1.58; 95% CI: 1.23-2.02; *p-value* = 0.002). The authors discussed the biological plausibility of these results exploring the differences between the intestinal flora of children who did not have contact with the maternal vaginal flora during birth, delaying acquisition of bifidobacterium and leading to changes in the composition of the intestinal flora that are associated with excess weight later on. In parallel, other researchers have analyzed the profiles of faecal samples from infants, finding that the intestinal microbiota of children with greater weight than expected had a different bacterial composition, some of which were species associated with protection against obesity at later ages [41].

The association between intestinal microbiota and a predisposition to obesity is a recent subject in the literature. First described in experimental studies with rats, the “energy product” hypothesis suggests that energy expenditure may be increased by use of indigestible carbohydrates by enzymes produced by the microbiota or that microbial metabolites exercise an influence on mechanisms that regulate their hosts intake and deposition of energy. However, the diversity of factors that influence the formation of microbiota, which include age, diet, lifestyle, ethnicity and genetics, combined with technical and methodological differences between intestinal flora analyses are challenges for studies that are ongoing. Currently, the question of whether changes to intestinal microbiota are a cause of obesity or a consequence of the disease remains unanswered [42].

Barros et al. [8] analyzed three Brazilian cohorts, but, after analyzing the prevalence of obesity in people aged 4, 11, 15 and 23 years who had been born by caesarean delivery, and after adjustment for confounding factors, the variable only had significance in one cohort and only for children aged 4. That study questions the role of alterations to intestinal flora as a cause of obesity, citing the lack of evidence to support the hypothesis and raising other possibilities, such as the presence of possible residual confounding factors; higher maternal educational level for example.

It is interesting to mention that the literature has shown an increase in the risk of caesarean delivery in overweight or obese women, compared to those with a normal weight [43]. Moreover, caesarean delivery reduces breastfeeding in the first hour of life [44], which may complicate the management of lactation later on. Therefore, the influence of these factors on the association between caesarean delivery and childhood obesity should be considered.

### Breastfeeding

Many studies have shown that prevention of diseases over the course of life is directly related to the nutrition received during gestation and the first years of life [9,10,13].

Breastfeeding has been seen as a protective factor against obesity, including some evidence showing that this protection increases with the time spent breastfeeding [6,12-16]. On the other hand, some authors did not find this protective association between breastfeeding and obesity over the life course [45]. An interesting finding of De Jesus et al. [39], while researching the same sample as our study, found that exclusive breastfeeding in the first four months is protective against overweight at four years of age; however, during the construction of this study, the association between exclusive breastfeeding for the first four months or six months with overweight and obesity at six years was tested and there was no statistical significance between the different groups. These data may suggest that the protective factor of breastfeeding can be reduced over time due to other predictors such as genetics or lifestyle factors.

The finding that caesarean delivery loses statistically significance as a risk factor in the group of children breastfed for at least 12 months raises the hypothesis that even though the aggression (caesarean delivery) occurred during a susceptible period of development, the physiological and metabolic system was adjusted, by means of breast milk, reprogramming those infants who did not have contact with their mothers’ vaginal microbiota during birth.

### Final comments

This research suffers from the methodological limitations inherent to prospective studies, with their susceptibility to follow-up losses, which in this case totalled 12 children lost to follow-up from the 684 children estimated in the sample size calculation. On the other hand, some methodological limitations that can be observed in other studies were not repeated in this investigation, since data were collected prospectively during home visits in which the children’s caregivers answered a 24-hour food recall. Additionally, the same technical team conducted all fieldwork from study outset to completion.

## Conclusions

Maternal obesity and caesarean delivery were strongly associated with childhood overweight/obesity. A lower family income and lower level of education were identified as protective factors. Breastfeeding appears to modify the association between overweight/obesity and the predictors studied. This study highlights the necessity for further research to investigate the association between caesarean delivery and childhood obesity while considering the role of possible confounders and residual effect modifiers.

These investigations must consider the need to stratify analyses by type of feeding, since risk factors for obesity are different among breastfed children than among children who were not breastfed or were weaned prematurely.
